# Detection of Plasmid-Mediated *qnr* Genes Among the Clinical Quinolone-Resistant *Escherichia coli* Strains Isolated in Tehran, Iran

**DOI:** 10.2174/1874285801812010248

**Published:** 2018-07-31

**Authors:** Reza Ranjbar, Sajjad S. Tolon, Mehrdad Sami, Reza Golmohammadi

**Affiliations:** 1 Molecular Biology Research Center, Systems Biology and Poisonings Institute, Baqiyatallah University of Medical Sciences, Tehran, Iran; 2 Department of Clinical Sciences, School of Veterinary Medicine, Ferdowsi University of Mashhad, Mashhad, Iran

**Keywords:** Antibiotic resistance, *E. coli*, *qnr*, PCR, Clinical strains, UTIs

## Abstract

**Background:**

*Escherichia coli* is one of the most important bacterial agents to cause urinary tract infections. Inappropriate and unnecessary administration of antibiotics has led to an increase in the appearance of multidrug-resistant *E. coli* isolates, limiting treatment options. The increase in a number of resistant strains of bacteria is a major concern of health authorities worldwide.

**Objective:**

The purpose of this study was to determine the presence of the *qnr* genes among *E. coli* isolated from UTIs of patients in Baqiyatallah hospital in Tehran province, Iran.

**Method:**

Clinical urine samples of patients with suspected urinary tract infection were collected by standard methods in sterile disposable containers. After analysis of urine, microscopic observations and culture analysis, the bacterial genome was extracted by boiling method. PCR for detection of *qnr* genes including *qnrA*, *qnrB* and *qnrS* was done by specific primers, then PCR products were run using gel electrophoresis and visualized by gel documentation system.

**Results:**

In the present study among the 95 isolates, 60 strains were resistant to nalidixic acid. PCR showed that 92 strains were positive for *qnrS*. The *qnrA* and *qnrB* genes were not found among the clinical isolates.

**Conclusion:**

Our finding indicates a high level of resistance against nalidixic acid among *E. coli* isolates recovered from the patients with UTI. Also, the high frequency of *qnrS* imposes the importance of survey of molecular and genetic analysis of mechanisms of quinolone resistance in *E. coli* strains.

## 1 INTRODUCTION

*Escherichia coli (E. coli)* is a commonplace bacteria that has been isolated from different sources [1, 2]. *E. coli* is an important gram-negative bacteria with the potential to cause serious disease such as Urinary Tract Infections (UTIs) [3]. UTIs, which are recognized as the most commonly reported nosocomial infections, account for up to 35% of infections associated with the health-care system, and *E. coli* bacteria is the most important cause of UTIs [4]. More than thirty patterns for pyelonephritis and cystitis [5] and variety of virulence genes among uropathogenic *E. coli* strains [6] were obtained. Due to the widespread resistance to other antibiotics, fluoroquinolones that are synthetic and broad-spectrum antibacterial agents often used for the treatment of UTIs in many countries [7-9].

The emergence of various isolates of the drug-resistant *E. coli* occurs due to the inappropriate and unnecessary administration of these antibiotics and leads to limiting the treatment options. In a recent study, Ranjbar et al. showed highly (96%) antibiotic resistance on clindamycin and frequency of different ESBLs genes among *E. coli* strains in the surface water sources [10]. Some studies indicated that urine specimens from isolates of different strains of *E. coli* have high resistance to one or more than one antibiotics [11-13]. Plasmid profile distinguished more strains than the antimicrobial susceptibility pattern [14]. The increase in a number of resistant strains of bacteria is a major concern of health authorities worldwide. Resistance to quinolone is mediated by different routes including mutations in DNA gyrase and topoisomerase subunits [15], decreased outer membrane permeability through porin changes and overexpression of naturally occurring efflux [16]. Moreover, mobile genetic elements, such as plasmids, play a role in quinolone resistance. The plasmid-mediated quinolone resistance is mediated by the genes (*qnr*) encoding proteins [17]. The major groups of *qnr* consist of *qnrA*, *qnrB* and *qnrS* [18, 19]. Plasmid-mediated resistance is growing clinical concern due to the transfer of resistance genes through the transfer of the horizontal gene to other species, conferring resistance against these antibiotics [20]. In addition, the presence of Extended-spectrum Beta-lactamases (ESBLs), AmpC, and *qnr* genes in a plasmid is well documented, indicating the complexity of the determinant factors involved in plasmid resistance among enterobacterial isolates in medical environments [21]. Obviously, the broad emergence of the growing trend associated with the prevalence of plasmid resistance in enterobacterial isolates is undeniable, however, only a limited number of studies of Iran about the prevalence of *qnr* gene among clinical isolates *E. coli* has been reported. The purpose of this study was to determine the presence of *qnr* genes among *E. coli* isolated from UTIs of patients in Baqiyatallah hospital in Tehran province, Iran.

## 2 MATERIALS AND METHODS

Over a period of one year, 600 patients that suspected urinary tract infections were examined for *E. coli* strains. Individual isolates were analyzed by standard biochemical and serological tests as described previously [22]. The slide agglutination test (Mast Diagnostics, Bootle, UK) was used for confirmation of the serogrouping of the isolates. Isolates were stored in trypticase soy broth (Merck KGaA, Darmstadt, Germany) containing 25% glycerol for future tests.

Antimicrobial susceptibility to nalidixic acid and ciprofloxacin was determined by using the disk diffusion method according to the Clinical Laboratory Standards Institute guidelines [23]. Antibiotic discs were placed within a distance of 12 mm from each other and incubated at 37ºC for 18 hrs. *E. coli* ATCC 25922 was used as quality-control strains to ensure the accuracy of the results obtained by susceptibility testing.

DNA was extracted using the AccuPrep® genomic DNA extraction kit (Bioneer, South Korea) according to the manufacturer’s instructions. The DNA concentration was determined by measuring absorbance of the samples at 260 nm using a spectrophotometer. The PCR and specific primers (Table **1**) used for the detection of *qnr*A, *qnr*B and *qnr*S plasmid-mediated quinolone-resistance genes [24]. PCR reaction was performed in a DNA thermocycler as follows: Initial denaturation at 96ºC for 5 min, 30 cycles of denaturation at 95ºC for 1 min, annealing at 55ºC for 1 min (based on the type of primer for each gene is different from that given in Table **1**), elongation at 72ºC for 1 min and a final extension step of at 72ºC for 7 min, followed by a hold at 4ºC. PCR products were electrophoresed on 1.5% agarose gel containing ethidium bromide at 80 V for 1 h [25].

## 3 RESULTS

**Fig. (1):**
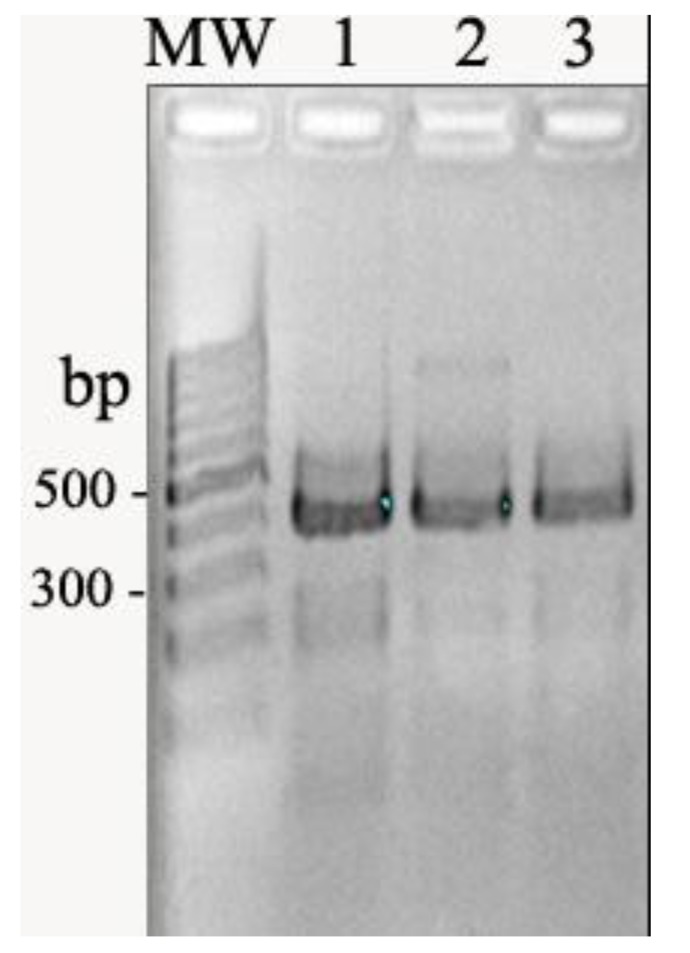
PCR amplification of qnrS genes in nalidixic acid-resistant Escherichia coli isolates.

Totally, 95 *E. coli* strains were isolated and enrolled in this study. Among the isolates, 60 and 49 strains were resistant to nalidixic acid and ciprofloxacin, respectively. PCR showed that 92 *E. coli* isolates carried *qnrS*. The *qnrA* and *qnrB* genes were not found among the clinical isolates of this study (Fig. **1**).

## 4 DISCUSSION

This study showed a high level of resistance against nalidixic acid among *E. coli* isolates recovered from the patients with UTI. Among the 95 isolates, 60 strains were resistant to nalidixic acid. Firoozeh *et al.* [26] showed that 82.5% and 45% of urinary *E. coli* isolates were resistant to nalidixic acid and ciprofloxacin, respectively. The resistance of *E. coli* isolates from nosocomial infections was 76% and 52%, to nalidixic acid and ciprofloxacin, respectively in Khorvash *et al.* study [27]. In China, the prevalence of ciprofloxacin resistance among *E. coli* isolated from urine was 59.4% [28]. Due to high rates of antibiotic resistance in this study and other studies, a full review of hospital management and further evaluation of monitoring systems are required. This study showed a high prevalence rate of plasmid-mediated quinolone resistance (*qnrS*). We did not find the *qnrA* and *qnrB* genes in our clinical isolates. The frequency of *qnrS* genes in our study was higher and the frequency of *qnrA* and *qnrB* genes in this study that was lower than those found in the two studies previously conducted in Iran. In another study, a low frequency (only a single *E. coli* isolate among 144 ciprofloxacin-resistant isolates) for *qnr* gene isolation was also described [29]. In Singapore [30] and Denmark [31], the nalidixic acid-resistant *E. coli* isolates as *qnr* positive was 1.8% and 1.6%, respectively. In Canada, only about 1% of *E. coli* and *Klebsiella* spp. isolates were resistant to ciprofloxacin and/or tobramycin as *qnr* positive [32]. Currently, resistance against quinolones and *qnr* genes has increased in many parts of the world including Iran among *Shigella* [33], *Salmonella* [34] and *E. coli* [35, 36] isolates. Uropathogenic *E. coli* is the most common cause of urinary tract infections, and quinolones resistant strains cause growing concern in developing countries [37]. Also in a recent study, the low frequency of *qnr* genes observed among the clinical isolates of *E. coli* in Iran [40-42]. In this study, *qnrS*-positive isolates showed high-level resistance, however other mechanisms such as secondary changes in DNA gyrase or topoisomerase IV, and porin or efflux systems, which was not evaluated in our study that could be involved in resistance patterns.

## CONCLUSION

In conclusion, our finding showed high frequencies of quinolone resistance genes among *E. coli* isolated from UTIs of patients in Baqiyatallah hospital in Tehran province, Iran. The circulation of strains of *E. coli* with a resistance plasmid gene can be considered as a risk for the spread of these types of genes among other bacteria, which requires special considerations. Also, the appropriate use of antibiotics may be useful to limit the potential spread of these resistant genes.

## Figures and Tables

**Table 1:** Primers used for detection of *qnr* genes in urinary *Escherichia coli* isolates.

Primer/target Amplicon	Primer Sequence (5´→3´)	Size of Product (bp)	Annealing Temperature (^◦^C)	Reference
*qnr*S	F: ACGACATTCGTCAACTGCAA	417	53	[38]
	R: TAAATTGGCACCCTGTAGGC			
*qnr*A	F: ATTTCTCACGCCAGGATTTG	516	53	[38]
	R: GATCGGCAAAGGTTAGGTCA			
*qnr*B	F: GTTGGEGAAAAAATTGACAGAA	383	53	[39]
	R: ACTCCGAATTGGTCAGATCG			
